# Supplementary material for: Brain-wide dynamics linking sensation to action during decision-making
Source: Nature. 2024 Sep 11;634(8035):890–900. doi: 10.1038/s41586-024-07908-w (PMC11499283; doi:10.1038/s41586-024-07908-w)
Supplement: Supplementary file 1 — Supplementary Tables 1 and 2 [file 41586_2024_7908_MOESM1_ESM.pdf]

nature portfolio

https://doi.org/10.1038/s41586-024-07908-w

Supplementary information

Brain-wide dynamics linking sensation to action during decision-making

In the format provided by the
authors and unedited

Supplementary Tables:

Area	# Mice	# Sessions	# Neurons (*# TF responsive*)
**SCs**	3	8	81 (*37*)
**LGd**	3	3	51 (*15*)
**VISp**	8	16	298 (*40*)
**LP**	5	15	415 (*72*)
**VISl/pl**	4	5	96 (*34*)
**PPC (VISa, VISam, VISrl)**	6	11	206 (*19*)
**RSP**	6	12	139 (*10*)
**CP**	9	31	1096 (*61*)
**GPe**	3	3	66 (*18*)
**SNr/GPi**	6	9	110 (*18*)
**LS**	2	3	80 (*0*)
**FRP**	3	7	86 (*4*)
**MOs**	9	31	1311 (*64*)
**ACA**	6	9	335 (*24*)
**mPFC (PL, ILA)**	5	10	319 (*19*)
**ORB**	4	13	976 (*29*)
**MOp**	6	15	700 (*12*)
**AI**	2	2	125 (*2*)
**MOB**	2	3	92 (*0*)
**DP**	4	8	165 (*3*)
**TTd**	3	7	119 (4)
**LD**	5	8	78 (*7*)
**CL**	4	7	116 (*8*)
**MD/VM**	5	9	102 (*9*)
**PF**	3	3	78 (*14*)
**VAL**	4	10	230 (*17*)
**PO**	5	8	308 (*17*)
**VB (VPL, VPM)**	5	11	366 (*30*)
**MG**	4	4	78 (*4*)
**RT**	3	9	280 (*12*)
**Eth**	5	5	87 (*4*)
**IC**	5	11	139 (*0*)
**MRN**	6	19	505 (*83*)
**APN**	3	7	134 (*32*)
**SCm**	4	13	312 (*64*)
**NPC**	1	2	51 (*11*)
**CA1**	7	27	229 (*12*)
**CA3**	7	14	179 (*14*)
**DG**	8	23	288 (*36*)
**SUB (POST, PRE, ProS, SUB)**	6	16	279 (17)
**ENT**	1	1	50 (*5*)
**Lob4/5**	6	21	596 (*39*)
**SIM**	5	19	533 (*33*)
**CRUS1/2**	4	5	188 (*9*)
**CENT3**	4	6	114 (*5*)
**DCN (FN, IP, DN)**	4	13	125 (*10*)
**FL/PFL**	3	3	47 (*1*)
**LHA**	3	6	136 (*1*)
**GRN**	1	3	137 (*9*)
**MV**	4	9	74 (*0*)
**Orofacial Nuc. (IRN, SPV, V)**	5	6	67 (*0*)

(Previous page) **Supplementary Table 1: Number of mice, sessions and neurons recorded per area, in trained mice.** Major brain region groups are designated by colour on the lefthand side. Brain region groups from top to bottom are: Early visual, Higher visual, Basal ganglia, Frontal cortex, Olfactory areas, Thalamus, Midbrain, Hippocampus, Cerebellum, Hypothalamus, Medulla.

Brain region name	*Abbreviation*
Anterior cingulate cortex	**ACA**
Anterior insular cortex	**AI**
Anterior pretectum nucleus	**APN**
Field CA1	**CA1**
Field CA3	**CA3**
Lobule 3	**CENT3**
Central lateral nucleus of the thalamus	**CL**
Caudoputamen	**CP**
Lobule Crus1	**CRUS1/2**
Deep cerebellar nuclei	**DCN**
Dentate gyrus	**DG**
Dorsal peduncular area	**DP**
Enthorinal cortex	**ENT**
Ethmoid nucleus of the thalamus	**Eth**
Flocculus/Paraflocculus	**FL/PFL**
Frontal pole	**FRP**
Globus pallidus, external part	**GPe**
Gigantocellular reticular nucleus	**GRN**
Inferior colliculus	**IC**
Infralimbic cortex	**ILA**
Interposed nucleus	**IP**
Lateral dorsal nucleus of the thalamus	**LD**
Lateral geniculate nucleus (dorsal part)	**LGd**
Lateral hypothalamic area	**LHA**
Lobule 4/5	**Lob4/5**
Lateral posterior nucleus of the thalamus	**LP**
Lateral septal nucleus	**LS**
Medio-dorsal nucleus	**MD**
Medial geniculate	**MG**
Main olfactory bulb	**MOB**
Primary motor cortex	**MOp**
Secondary motor cortex	**MOs**
Medial prefrontal cortex	**mPFC**
Midbrain reticular nucleus	**MRN**
Medial vestibular nucleus	**MV**
Nucleus of the posterior commissure	**NPC**
Olfactory nuclei	**OLF**
Orbital cortex	**ORB**
Orofacial Motor Nuclei	**Orofacial Mot. Nuc./OF. Nuc**
Prelimbic cortex	**PL**
Lateral visual cortex/Posterolateral visual cortex	**VISl/pl**
Primary visual cortex	**VISp**
Parafascicular nucleus	**PF**
Posterior nucleus of the thalamus	**PO**
Postsubiculum	**POST**
Posterior parietal cortex	**PPC**
Retrosplenial cortex	**RSP**
Thalamic reticular nucleus	**RT**
Superior colliculus, motor part	**SCm**
Superior colliculus, sensory part	**SCs**
Simplex lobule	**SIM**
Spinal trigeminal (interpolar part)	**SPVI**
Spinal trigeminal (oral part)	**SPVO**
Primary somatosensory cortex	**SSp**
Globus pallidus, internal part	**SNr/GPi**
Subiculum	**SUB**
Taenia tecta, dorsal part	**TTd**
Ventral anterior-lateral complex of the thalamus	**VAL**
Ventrobasal complex of the thalamus	**VB**
Ventral medial/medial dorsal nuclei of the thalamus	**VM/MD**
Ventro-medial nucleus	**VM**
Anterior visual cortex	**VISa**
Anterio-medial visual cortex	**VISam**
Lateral visual cortex	**VISl**
Postero-lateral visual cortex	**VISpl**
Rostro-lateral visual cortex	**VISrl**
Ventral postero-lateral nucleus	**VPL**
Ventral postero-medial nucleus	**VPM**

Supplementary Table 2: List of brain region abbreviations
